# Deep reinforcement learning and robust SLAM based robotic control algorithm for self-driving path optimization

**DOI:** 10.3389/fnbot.2024.1428358

**Published:** 2025-01-15

**Authors:** Samiullah Khan, Ashfaq Niaz, Dou Yinke, Muhammad Usman Shoukat, Saqib Ali Nawaz

**Affiliations:** ^1^College of Electrical and Power Engineering, Taiyuan University of Technology, Taiyuan, China; ^2^Hubei Key Laboratory of Advanced Technology for Automotive Components, School of Automotive Engineering, Wuhan University of Technology, Wuhan, China; ^3^School of Information and Communication Engineering, Hainan University, Haikou, China

**Keywords:** autonomous navigation, robotic control, path tracking, deep reinforcement learning, SLAM, RS-DDPG algorithm

## Abstract

A reward shaping deep deterministic policy gradient (RS-DDPG) and simultaneous localization and mapping (SLAM) path tracking algorithm is proposed to address the issues of low accuracy and poor robustness of target path tracking for robotic control during maneuver. RS-DDPG algorithm is based on deep reinforcement learning (DRL) and designs a reward function to optimize the parameters of DDPG to achieve the required tracking accuracy and stability. A visual SLAM algorithm based on semantic segmentation and geometric information is proposed to address the issues of poor robustness and susceptibility to interference from dynamic objects in dynamic scenes for SLAM based on visual sensors. Using the Apollo autonomous driving simulation platform, simulation experiments were conducted on the actual DDPG algorithm and the improved RS-DDPG path-tracking control algorithm. The research results indicate that the proposed RS-DDPG algorithm outperforms the DDPG algorithm in terms of path tracking accuracy and robustness. The results showed that it effectively improved the performance of visual SLAM systems in dynamic scenarios.

## 1 Introduction

At present, self-driving technology is one of the research hotspots in the field of artificial intelligence, and reinforcement learning (RL) has drawn significant attention in recent years. In self-driving systems, the most fundamental challenge is the control of path tracking (Yuan et al., 2021). The goal of path tracking control is to enabling vehicles to travel along a predetermined path and approach the fixed trajectory as closely as possible. SLAM is one of the rapidly developing robot perception technologies in recent years, which has been applied in fields such as autonomous navigation (Zheng et al., 2023), augmented reality (Shoukat M. U. et al., 2023), and medical equipment (Shoukat K. et al., 2023). Path tracking control methods are mainly divided into two categories: model based and non-model based. Among them, model-based path tracking control methods mainly rely on the kinematic and dynamic models of the robot, and control the robot’s motion by outputting control signals from the controller. Common model-based control methods include proportional integral differential (PID) control (Arrieta et al., 2023; Gopi Krishna Rao et al., 2014; Huba et al., 2023), fuzzy control (Benbouhenni et al., 2023; Zhang et al., 2023), model predictive control (MPC; Fiedler et al., 2023; Wei and Calautit, 2023; Bayat and Allison, 2023), etc. Non-model based path tracking control methods do not require an accurate robot model, but control is achieved through perception and decision-making modules. Common non-model based control methods include neural network control methods (Legaard et al., 2023; Sun et al., 2023).

In the past, researchers have sought solutions from model-based algorithms to address issues such as low tracking accuracy and poor robustness of AVs towards target paths during operation. Mendoza and Yu (2023) proposed a fuzzy adaptive PID control method based on vehicle kinematics and dynamics is used to plan the next driving path based on preview theory. They first used the position relationship between the vehicle center of mass and the desired path preview point to calculate the lateral deviation and heading deviation, and then used the fuzzy adaptive PID controller to adjust the front wheel angle by adjusting the error. Although this method is simple and feasible, its adaptability and control accuracy are limited in high demand control situations. Chen et al. (2023) proposed a horizontal and vertical fuzzy control method based on dynamic dual point preview strategy, which dynamically controls the dual point preview distance through fuzzy control, thereby controlling the vehicle to track the corresponding trajectory. However, the effectiveness of fuzzy control is greatly affected by changes in the preview distance. Wu et al. (2023) proposes a composite fuzzy control method based on lateral error and heading error. This method adjusts the output of two fuzzy controllers by specifying corresponding weight variables, and uses integral compensation to solve the problem of low steady-state accuracy in traditional fuzzy control. However, this method has poor path tracking accuracy in complex roads.

In order to ensure the stability and overall performance of the control-loop system, Roman et al. (2024) suggested a data-driven approach that combines an algorithm with continuous-time active disturbance rejection control. Nguyen et al. (2023) proposed a control algorithm for vehicle trajectory tracking using linear time-varying MPC. Compared to nonlinear control, this method has a global optimal solution and smaller computational complexity. Nevertheless, this method requires high modeling requirements for vehicles, linear approximation for nonlinear systems, and the construction of a quadratic cost function. At the same time, this method requires high hardware storage space and computing power, and needs to consider the limitations of computing resources appropriately. Xue et al. (2024) suggested a novel control technique, which utilizes a neural network (NN) and policy iteration (PI) algorithm to achieve H∞ control in a nonlinear system. Jing (2024) introduced a NN modelling method that utilizes evolutionary computation (EC). The method includes techniques such as NN model compression, distributed NN model, and knowledge distillation. Although the control algorithm can stably track the path, but the system is too complex and has poor stability performance. Chi et al. (2022) presented a method for improving the P-type controller using set point learning called indirect adaptive iterative learning control to improve both linear and nonlinear systems. Zhou et al. (2023) adopts a combination of neural network and fuzzy control method to control the driving direction of the vehicle by controlling the steering wheel angle. The control effect of this method is relatively stable, but there are problems such as untimely steering control and large tracking errors. Model based control algorithms in path tracking rely on robot models, and robot modeling is a complex process that not only needs to consider the influence of various factors such as mechanical structure, dynamic characteristics, control strategies, etc., but also needs to consider the influence of various uncertain factors, making modeling difficult. There are primarily three problems in raising and ensuring the proper functioning of the intelligent vehicular path optimization system.

- Model based control algorithms have a high degree of dependence on the model in the path tracking process, but robot modeling is difficult, which can lead to poor tracking accuracy.
- Non model based control algorithms require a large amount of robot and environmental data for neural network learning, and the completeness of environmental data collection is difficult to meet, which leads to poor tracking performance.
- Current technical conditions are difficult to ensure the integrity of environmental data collection. Lack of complete environmental data can lead to inaccurate information learned by neural networks, resulting in poor tracking performance.

An integrated method combining DRL’s adaptability for learning optimal pathways and SLAM’s robustness for reliable localization and mapping is necessary to address these problems. To fill in data gaps, the suggested system fuses inputs from several sensors (e.g., LiDAR, radar, and cameras) and using SLAM to produce a continuous, real-time environment map. Table 1 shows the advantages, disadvantages, and main application scenarios of several common visual sensors.

**Table 1:** Sensor performance.

Sensor type	Advantages	Disadvantages	Application scenarios
LiDAR (Li et al., 2024)	Good robustness and high stability; Low computational complexity and lower CPU requirements than camera sensors	Unable to obtain semantic information; Not suitable for harsh environments, such as rainy and foggy weather; Unable to obtain depth information of the perspective body	Indoor low-speed small-scale scenes
Monocular camera (Yu et al., 2023)	Simple structure; Low cost; The calibration and identification process are easy	Unable to determine the depth information of individual images and the true size of objects	Indoor and outdoor scenes
Binocular camera (Zhang et al., 2024)	Can determine the true scale of an object	Large computational load; The calibration process is complex; GPU or FPGA acceleration is required, which consumes a huge amount of computing power	Indoor and outdoor small-scale scenes
RGB-D camera (Song et al., 2023)	Strong dynamism and low computational complexity	Narrow field of view, small measurable range, easily affected by light, unable to recognize transmissive objects	Indoor small-scale scenes
Event camera (Messikommer et al., 2022)	Low latency; Low computational power consumption and low computational power requirements; High dynamic range	Strong data sparsity; More redundant information, less effective information	High speed and high dynamic scenes

From Table 1, it can be seen that the visual SLAM using cameras as sensors has gradually become one of the main research directions in the field of SLAM. Yuan et al. (2023) calculated the transformation matrix between two frames based on the results of feature point matching and then used this matrix to extract line features and evaluate their static weights. Finally, the remaining static features were used for camera pose estimation to complete the tracking task. Montemerlo and Thrun (2003) proposed FastSLAM by combining EKF and RBPF algorithms. This algorithm estimates the robot pose using the RBPF algorithm and then updates the map using EKF, achieving accurate localization of the robot in unknown environments. Shoukat et al. (2024) introduced a graph SLAM system that utilized YOLOv5 and Wi-Fi fingerprint sequence matching. This algorithm aims to improve the accuracy and resilience of closed-loop detection for robot navigation. Zhang et al. (2018) improved the traditional SLAM system and proposed a dynamic object detection algorithm based on geometric constraints. Dai et al. (2020) used the Delaunay triangulation method to establish a structure similar to the graph for map points, in order to determine their adjacency relationship.

This balanced strategy improves tracking precision, data integrity, and system resilience under real-world uncertainty. This system could improve self-driving technology by improving navigation accuracy, reliability, and adaptability in changing surroundings. The algorithms for RL include SARSA (state action reward state action; Naderi et al., 2023), Q-learning (Puente-Castro et al., 2024), DQN (deep Q-network; Yang and Han, 2023), DDPG (Na et al., 2023), etc. SARSA first creates a Q table and updates its status through interaction with the environment, then takes actions based on the values in the Q table. However, SARSA can only target some simple games. Q-learning is similar to SARSA. The difference in Q-learning is that different strategies are chosen when updating the Q-table, but it is essentially in the form of a table. Q-learning selects the optimal strategy through the Q-table. Moreover, Tampuu et al. (2017), utilizes DQN to train individual agents in a two-player Pong game. However, considering other agents as part of the environment causes instability because agents may adjust their strategies independently. DQN is based on Q-learning and introduces neural networks instead of Q-tables to save software space, but it is not suitable for continuous spaces.

DDPG is a strategy that facilitates depth function approximation, which can be applied in high-dimensional and continuous spaces, while the first three algorithms are only applicable to low dimensional and discrete behavioral spaces. However, in high-dimensional, continuous action autonomous driving, the reward and penalty mechanism of DDPG cannot be well set. Based on the above analysis, it can be concluded that both models based and non-model based path tracking control algorithms have some shortcomings in the path tracking process. Within this particular context, the primary contributions of this paper can be summarized as follows:

- In response to the drawbacks of the above path tracking control algorithms, we developed a reward-shaping deep deterministic policy gradient (RS-DDPG) algorithm for path tracking control maneuvers. This algorithm does not rely on precise data models of the system or requires a large amount of environmental data.
- This article proposed a visual SLAM method for dynamic scenes by combining semantic segmentation networks and multi view geometry methods.
- RS-DDPG for continuous-action tasks in DRL framework to address optimization and robustness concerns. This approach promotes agent collaboration.
- In the proposed algorithm, the robot’s path tracking control is achieved by designing reward functions and adaptive weight coefficients based on factors such as the yaw deviation between the robot and its expected trajectory, the lateral angular velocity of the robot, and other relevant parameters.

## 2 Preliminary

### 2.1 Markov decision process

The essence of DRL is the interaction process between intelligent agents and the environment, which can be regarded as a Markov decision process (MDP). MDP is a time-dependent sequential decision-making process, where the state at the next moment depends only on the current state and action. MDP defines a five tuple $\left(S,A,R,P,\gamma \right)$ , where, $s=\left\{s_{1},s_{2},s_{3},\ldots \right\}$ represents the state of the robot; $A=\left\{a_{1},a_{2},a_{3},\ldots \right\}$ signifies the actions output by the intelligent agent in the current state; $R=\left\{r_{1},r_{2},r_{3},\ldots \right\}$ denotes the reward for the output action in the current state, with lag effect; $P=p\left[s_{t+1},r_{t}\;|s_{t},a_{t}\right]$ represents the probability function of $s_{t}$ output action at $a_{t}$ transferring to the next state $s_{t+1}$ and receiving reward $r_{t}$ in the current state; and γ is the discount factor, and $\gamma \in \left[0,1\right]$ .

This study specifies the state-action rate role for any policy π in a very large state-action space. Because getting an exact estimate of $Q_{\pi }\left(s,a\right)$ is not practicable, function approximations such linear functions and neural networks are used (Yang et al., 2022; Yang et al., 2022). Neural networks’ strong function approximation abilities have led to their extensive practical application across many domains.

### 2.2 Reinforcement learning process

**Figure 1:**
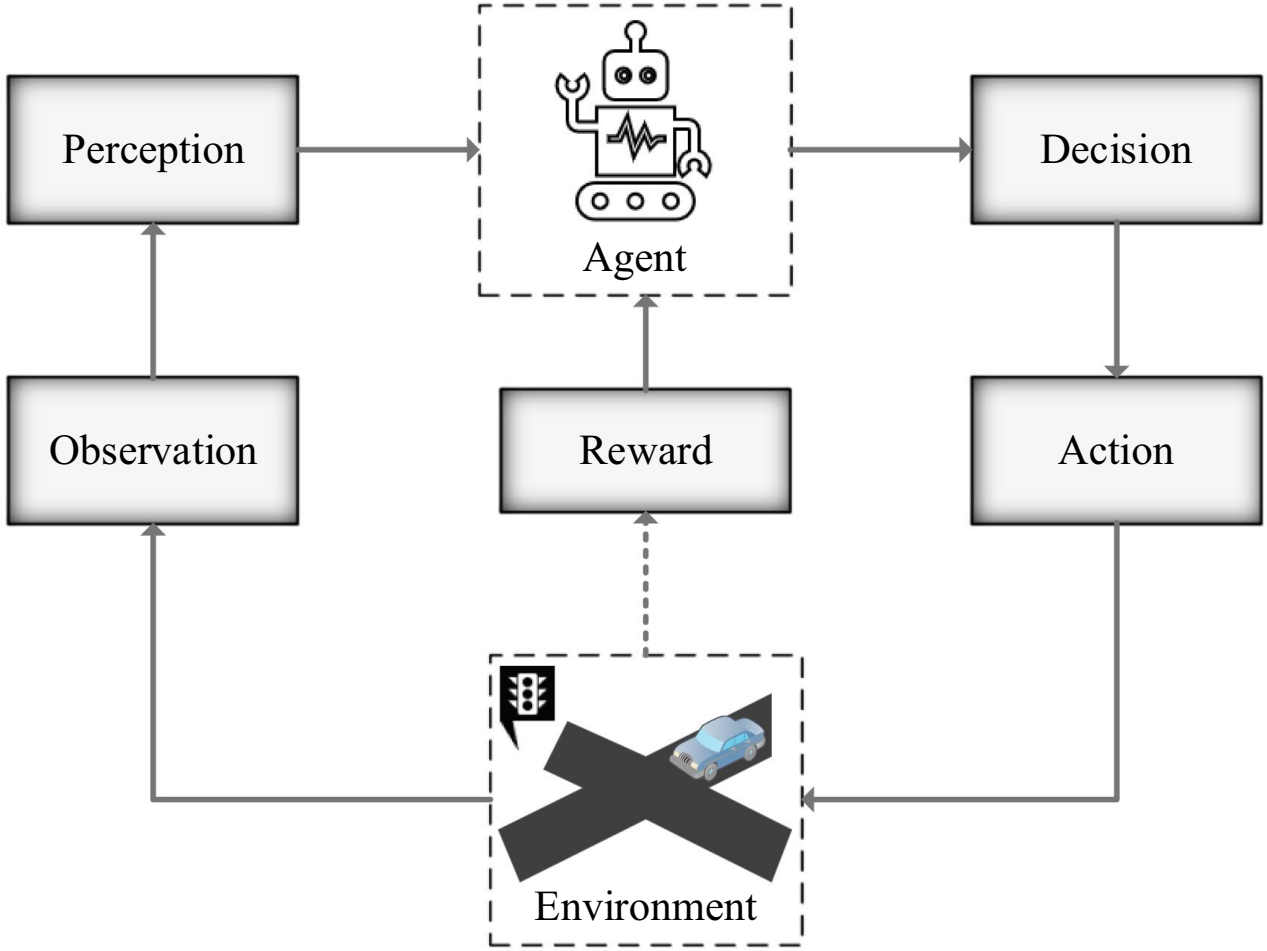
Schematic diagram of reinforcement learning process.

In the process of RL, the agent gives action A based on the current state parameter S at each time point, and then enters the next environmental state, providing feedback reward R (schematic diagram of RL process is shown in Figure 1). Then a series of data ( $s_{1},a_{1},r_{1},s_{2}.a_{2},r_{2},\ldots ,s_{t},a_{t},r_{t}$ ) will be recorded in the memory pool, and the cumulative return $G_{t}$ will be calculated using the following formula:


(1) $$G_{t}=R_{t+1}+\gamma R_{t+2}+\ldots =\overset{+\infty }{\underset{k=0}{\sum }}\gamma^{k}R_{t+k+1}$$

Use π to represent the policy of the intelligent agent $\pi \left(a,s\right)=p\left(A_{t}=a|S_{t}=s\right)$ , and select the probability of outputting action a based on the current state s . Use the value function Q to represent the value of action a taken by s in the current state as $Q\left(s,a\right)=E_{\pi }\left(G_{t}|A_{t}=a,S_{t}=s\right)$ . Where, $E\left(x\right)$ is the expected function. The value function obtained by recursion using the Bellman equation is as in Equation 2:


(2) $$Q\left(s,a\right)=E_{\pi }\left(R_{t+1}+\gamma Q\left(S_{t+1},A_{t+1}\right)|A_{t}=a,S_{t}=s\right)$$

The core task of RL is to continuously adjust strategies to maximize the value of the reward function. In the process of reinforcement learning, the agent updates the strategy by maximizing the value of the reward function. The strategy then gives the next action and receives the reward, which loops through to ultimately achieve the system control goal.

**Figure 2:**
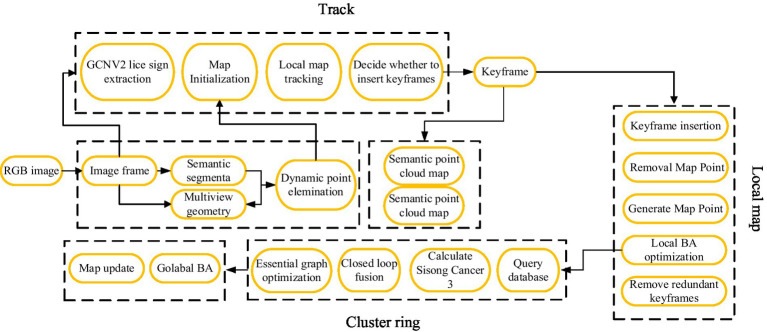
Overall framework of visual SLAM algorithm.

In the camera mode of SLAM, combined with semantic segmentation, a semantic segmentation module and a thread for constructing a semantic octree map are added on top of the original front-end odometry, local mapping, and loop detection threads. The overall framework is shown in Figure 2. Firstly, the RGB image obtained by the RGB-D camera is fed into the tracking thread. In the tracking thread, the GCNv2 network is used to extract the key points and descriptors of the current frame. Afterwards, pixel level semantic segmentation is performed on the RGB image through a semantic segmentation network to segment specific objects, including dynamic and static target objects, and preliminary removal of dynamic feature points, such as walking people, is performed. And combined with multi view geometric methods for detection (Cui and Ma, 2019), further removing dynamic objects, and using the remaining static features for pose estimation. Finally, in the semantic map construction thread, the semantic information extracted through semantic segmentation is used to generate a point cloud map and convert it into an octree map.

### 2.3 Feature extraction

**Figure 3:**
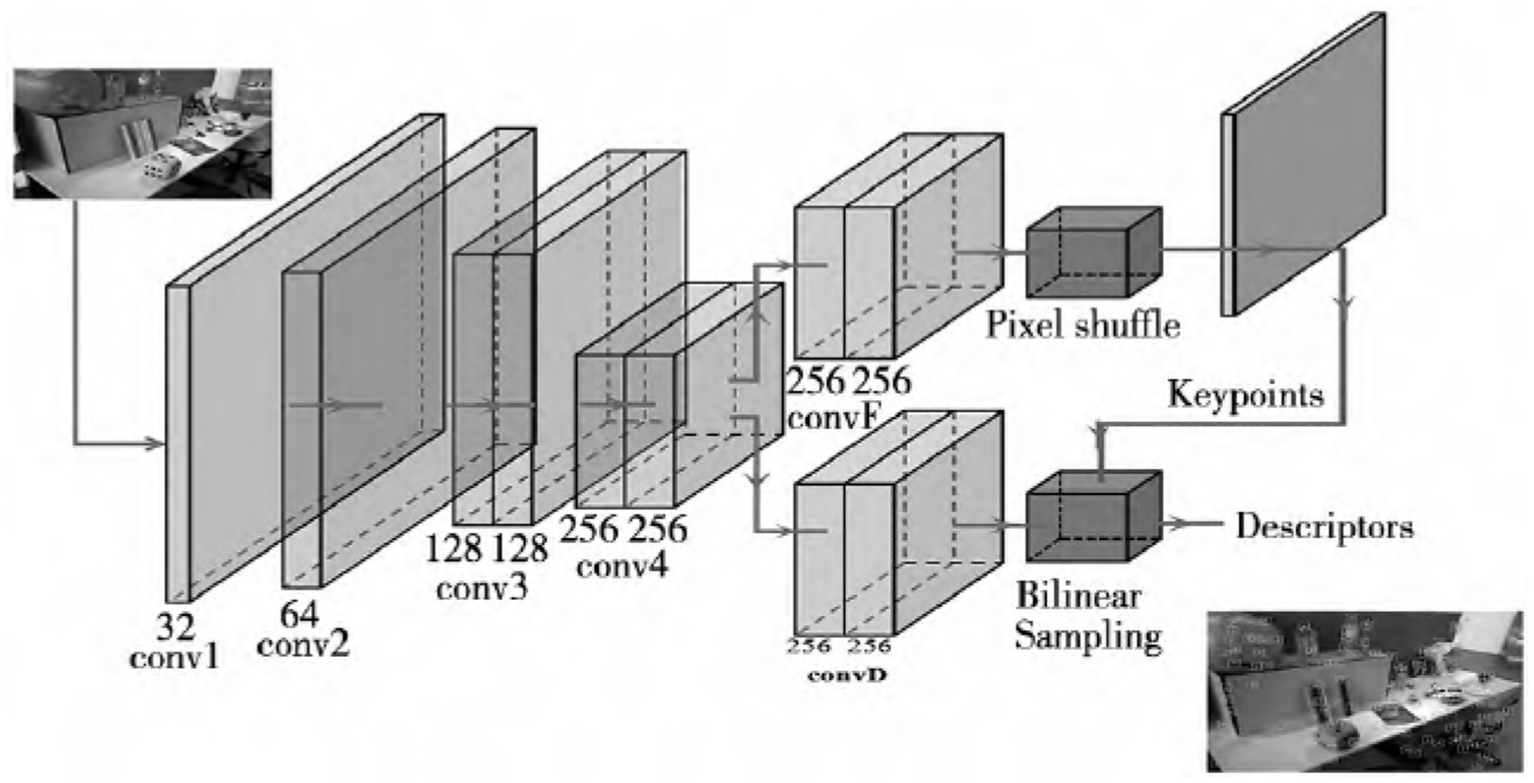
GCNv2 network structure.

The GCNV2 is a network trained for 3D projection geometry that can be used to extract feature points and descriptors. In contrast to the conventional approach of training with a single image, GCNv2 trains on the TUM and SUN-3D datasets using image pairs. In order to obtain feature points and their corresponding descriptors that are uniformly distributed, GCNv2 takes the input single-channel image and scales it to 320 × 240. The network then takes this adjusted image, extracts its features, and processes them using homogenization and non-maximum suppression. The GCNv2 method’s feature extraction procedure is shown in Figure 3 (Shao et al., 2022). It can clearly see from the graph that the extracted features are evenly distributed throughout the entire image.

### 2.4 Semantic segmentation network

**Figure 4:**
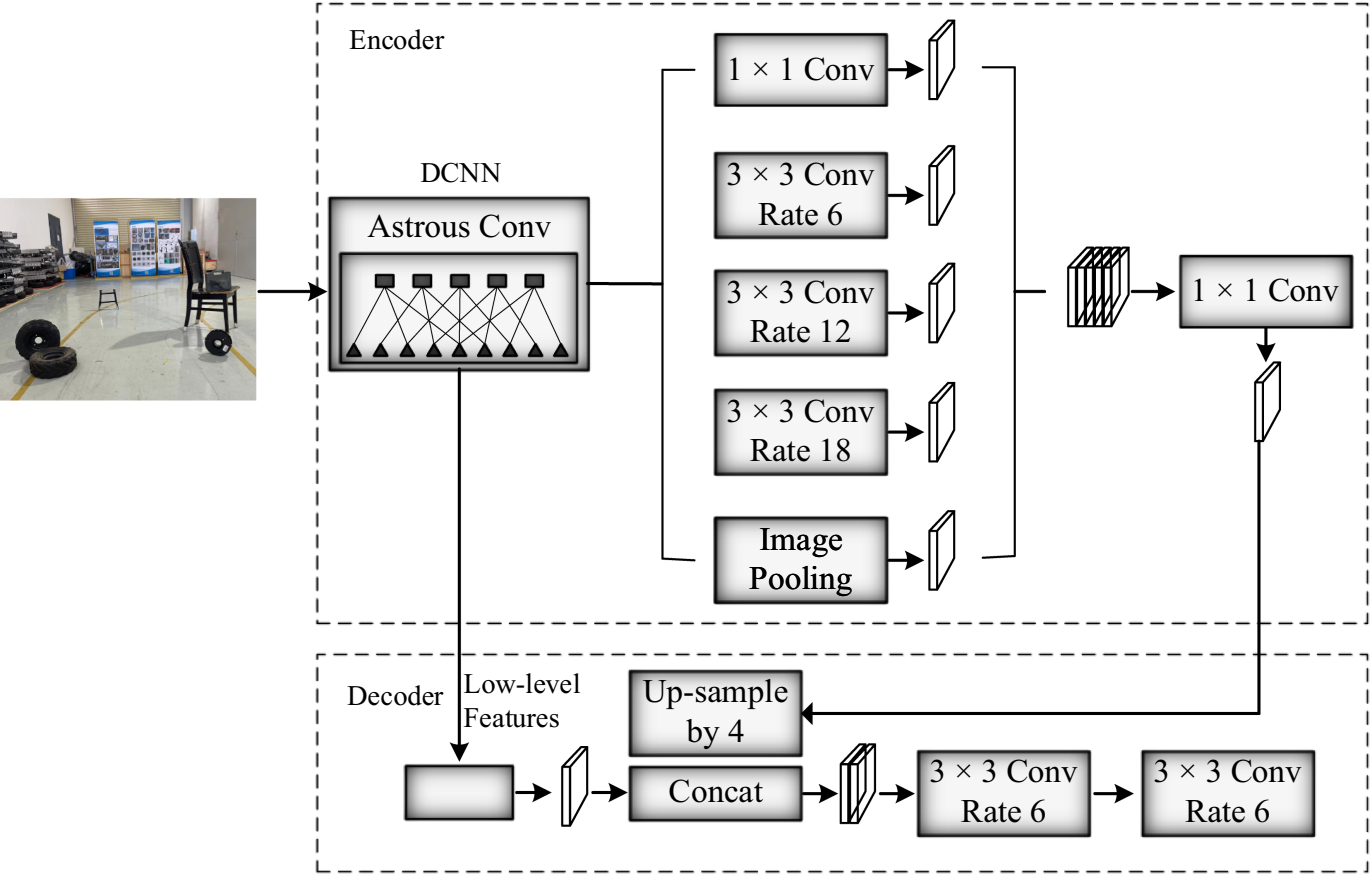
DeepLabv3+ semantic segmentation.

This paper uses the segmentation network DeepLabv3+ (Hu et al., 2022) to complete the semantic segmentation task of image frames. In recent years, many scholars have continuously proposed new semantic segmentation networks, such as PSPNet with a pixel accuracy of 0.9293 and BiSeNet with a pixel accuracy of 0.9337. DeepLabv3+ adds a decoder module to the framework of DeepLabv3, and integrates multi-scale information in the atrous spatial pyramid pooling layer (ASPP) module based on dilated convolution. In the decoder architecture, more accurate object boundaries are obtained through spatial information recovery, optimizing segmentation results, and achieving a pixel accuracy of 0.9431, so, this article chooses DeepLabv3+. Figure 4 shows the process of semantic segmentation algorithm, where pixels represent people and blue pixels represent display screens. After inputting the image into the network, two output values are obtained through DCNN feature extraction: feature map 1 containing high-level semantic information and feature map 2 containing low-level features. Map1 first passes through the ASPP module, and then utilizes 1 × 1 to adjusting the number of channels for convolution yields map1’. Map2 utilizes 1 × 1 to adjust the number of channels for convolution to obtain map2’. Perform 4 up-sampling operations on map1’ and concatenate it with map2’. Finally, use 3 × 3 for channel adjustment with 3 convolutions, the final segmentation result is obtained through quadruple up-sampling.

### 2.5 Semantic map construction

In the semantic map construction thread, PCL library is used to generate point clouds by combining keyframes and depth maps. Then, the pose of the current frame and its point cloud are used for point cloud stitching and filtering processing to generate a point cloud map, and semantic information is annotated in the point cloud map. However, although point cloud maps give people a very intuitive feeling, they have disadvantages such as occupying a large amount of storage space, redundant location information, and cannot be directly used for navigation. Compared to this, octree maps (Ju et al., 2020) also have the intuitiveness of point cloud maps, but their storage space is much smaller, making them suitable for various navigation purposes. Therefore, this article further processes point cloud maps by converting them into octree maps and constructing semantic octree maps based on semantic information. However, during the mapping process, due to camera noise and errors caused by dynamic objects, the same node may have different states at different time points. So, we use probability to explain whether a node is occupied or not. However, this method may result in a probability greater than 1, which can interfere with data processing. Therefore, the probability logarithm is used to describe whether a node is occupied. Let y∈R (real number set) represent the probability logarithm, and the range of occupied probability p is [0,1]. The logit transformation formula is $y=\log\left(p\right)=\log\left(\frac{p}{1-p}\right)$ . The reversible transformation for logit transformation is as in Equation 3:


(3) $$p=\log it^{-1}\left(y\right)=\left(\frac{1}{1+\exp\left(-y\right)}\right)$$

Assuming that the observation probability of a node n at time T is $P\;\left(n|Z_{t}\right)$ , where Z represents the observation data. The probability of its occupation $P\;\left(n|Z_{1:T}\right)$ is represented as in Equation 4:


(4) $$P\left(n|Z_{1:T}\right)=\left[1+\frac{1-P\left(n|Z_{T}\right)}{P\left(n|Z_{T}\right)}\frac{1-P\left(n|Z_{1:T-1}\right)}{P\left(n|Z_{1:T-1}\right)}\right]-\frac{P\left(n\right)}{1-P\left(n\right)}$$

where, $P\left(n\right)$ represents the prior probability of node n being occupied, and $P\left(n|Z_{1:T-1}\right)$ represents the estimated probability of node n from the beginning to the T−1 moment. In this article, we set the prior probability $P\left(n\right)$ to 0.5, and the above equation is transformed into a probability pair in the form of $L\left(n|Z_{1:T}\right)$ , which represents the logarithmic value of the probability of node n from the beginning to time T . Therefore, at time T+1 , it is as in Equation 5:


(5) $$L\left(n|Z_{1:T+1}\right)=L\left(n|Z_{1:T-1}\right)+L\left(n|Z_{T}\right)$$

here, $L\left(n|Z_{1:T+1}\right)$ and $L\left(n|Z_{T}\right)$ represent the logarithmic values of the probability of node n being occupied before and at time T . According to Equation 3, when a node is repeatedly observed and occupied, its probability logarithm increases, otherwise it decreases. Based on the obtained information, the occupancy probability of this node can be dynamically adjusted to continuously update the octree map.

**Figure 5:**
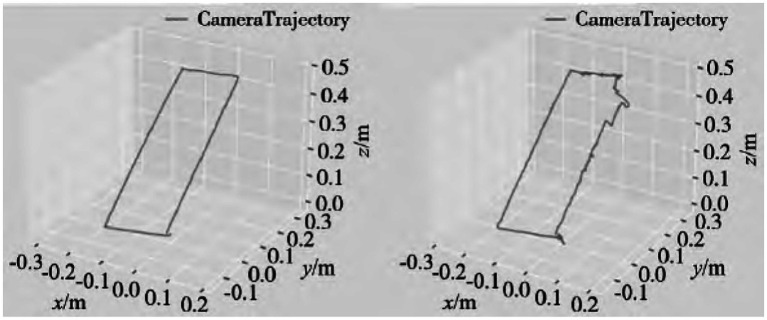
Comparison of motion trajectories: **(A)** Algorithm **(B)** ORB-SLAM2 algorithm in this article.

This article treats a moving person as a dynamic object, and uses a KinectV2 camera mounted on a mobile robot to move uniformly in a dynamic environment according to a previously designed rectangular path, perceive the surrounding environment, and collect information in the scene. Subsequently, using ROS tools, the obtained real scene data was split into frame images, and the TUM dataset was used as the standard to produce the obtained real scene data in the format of the TUM dataset. The algorithm proposed in this paper and the ORB - SLAM2 algorithm were tested to verify their effectiveness and feasibility. Figures 5A,B respectively represent the three-dimensional motion trajectories generated by our algorithm and ORB - SLAM2 algorithm. From Figure 5, it can be clearly seen that due to the presence of moving objects in the experimental scene, the trajectories generated by ORB - SLAM2 algorithm show significant fluctuations compared to the actual motion trajectories. However, the trajectories generated by our algorithm are basically consistent with the actual motion trajectories, and the fluctuation amplitude is relatively small.

This study uses GCNv2 for feature extraction, compared with traditional SLAM system feature extraction methods, the extracted feature points are more evenly distributed. The semantic segmentation network DeepLabv3+ is used to assign semantic information to the image frames in the visual SLAM system, detect moving targets in the objects, and then combine geometric information to detect dynamic feature points.

## 3 Reward shaping DDPG algorithm

### 3.1 DDPG algorithm

**Figure 6:**
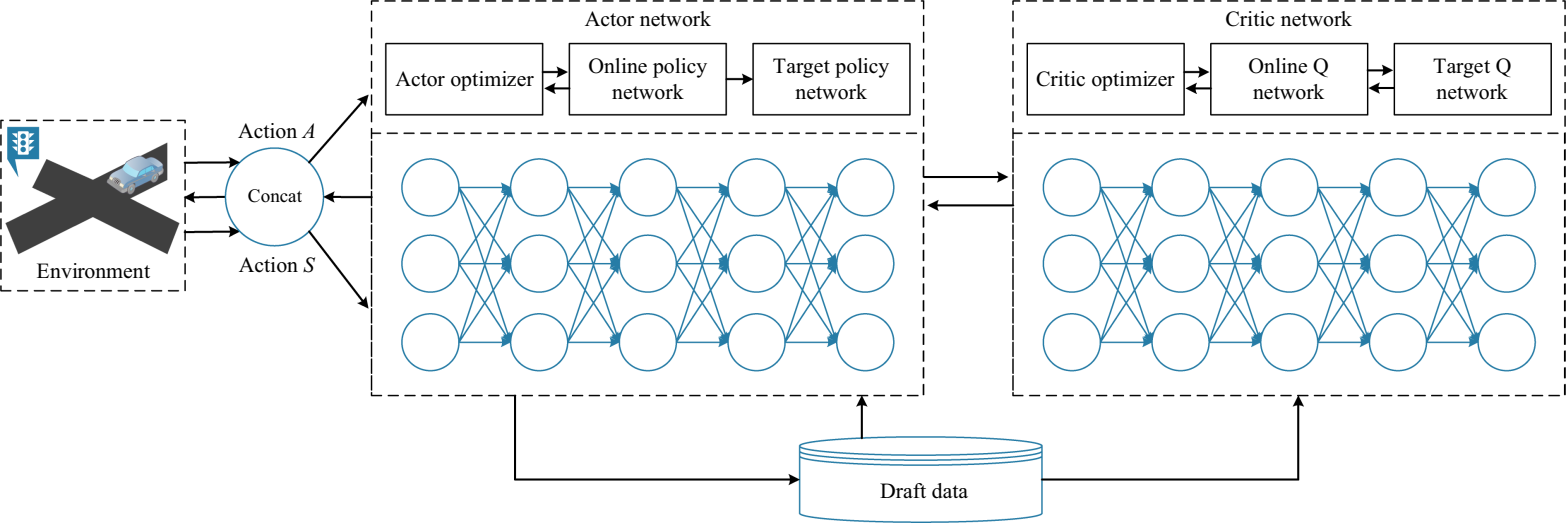
DDPG algorithm with actor and critic networks.

Among the many actor-critic algorithms that use neural network approximations, DDPG is among the most well-known. DDPG is a model shaping algorithm based on deterministic policy gradients, which is based on the actor-critic framework and can be applied to continuous behavior spaces. The actor network denoted as $\mu \left(s|\theta^{\mu }\right)$ , maps a state s to an action a using parameters $\theta^{\mu }$ . The critic network $Q\left(s,|a,|\theta^{Q}\right)$ , evaluating actions with a learning rate $\alpha_{Q}$ with parameters $\theta^{Q}$ . Training parameters like the total number of episodes and steps per episode establish the overall training duration. The function of the actor network is to output action A based on the state S feedback from the environment; The function of the critic network is to output the Q value based on the state S feedback from the environment and the corresponding action A of the actor. The function of actor target network and critic target network is to improve the stability of the network. The network first fixes its own parameters for a period of time, and then updates its own parameters by copying the parameters of the actor network and the critic network, as shown in Figure 6.

On the basis of state observation, the actor network outputs corresponding decision behaviors and parameterizes these behaviors into an n-dimensional vector θ with policy π is $\pi \left(a|s,\theta \right)=p\left(A_{t}=a|S_{t}=s\right)$ . The projected long-term return is estimated by a critic parameterized by ω in DDPG, while an actor parameterized by θ generates a deterministic policy $\pi_{\theta }$ .

The actor network is updated based on the policy gradient method, and the policy is improved through the policy gradient. The policy gradient $\nabla_{\theta }J\left(\theta \right)$ expression is represented in Equation 6:


(6) $$\nabla_{\theta }J\left(\theta \right)={\left[\frac{\partial J\left(\theta \right)}{\partial \theta_{1}}\;\frac{\partial J\left(\theta \right)}{\partial \theta_{2}}\ldots \frac{\partial J\left(\theta \right)}{\partial \theta_{n}}\right]}^{T}$$

where, $J\left(\pi_{\theta }\right)$ is the policy objective function. The policy gradient $\nabla_{\theta }J\left(\theta \right)$ expression for stochastic policies is $\nabla_{\theta }J\left(\pi_{\theta }\right)=E_{\pi \theta }\left(\left[\nabla_{\theta }\log\pi_{\theta }\left(s,a\right)\right]Q^{\pi }\left(s,a\right)\right)$ . $\nabla_{\theta }\log\pi_{\theta }\left(s,a\right)$ is a fractional function, which can be expressed in Equation 7:


(7) $$\nabla_{\theta }\log\pi_{\theta }\left(s,a\right)=\left(\nabla_{\theta }\pi_{\theta }\left(s,a\right)\right)/\left(\pi_{\theta }\left(s,a\right)\right)$$

In deterministic strategy, $a=\mu_{\theta }\left(s\right)$ , the gradient of deterministic strategy is a special form of stochastic policy where the gradient variance approaching 0 , and its gradient expression is as in Equation 8:


(8) $$\nabla_{\theta }J\left(\mu_{\theta }\right)=E_{\mu \theta }\left(\nabla_{\theta }\mu_{\theta }\left(s\right)Q^{\mu }\left(s,a\right)|\mu_{\theta }\left(S\right)\right)$$

In the learning process of intelligent robot trajectory tracking control, the input of the actor neural network is the observed environmental state variables, such as position, angle, speed, etc. Its output is decisions made based on strategies, such as steering wheel angle and throttle braking. At the same time, critic’s approach is based on the behavioral value function, where the input variables are state and behavior, and the output variables are return values. During the learning process, critic uses the estimated value function as the benchmark for updating the actor function, while evaluating the actor’s strategy. The advantage of the actor-critic method is that critic provides a more accurate evaluation through the value function, thereby improving the actor strategy and making it more optimized. In addition, the actor-critic method can not only use critic to update actor policies, but also update the value function of critic, which can better evaluate behavioral value.

In practice, the value function of critic is updated using the Bellman equation $Q^{\prime }\left(s,a\right)=Q\left(s,a\right)+\alpha \left[R\left(s,a\right)+\gamma \;\max Q\left(s,a\right)-Q\left(s,a\right)\right]$ , where, α is for learning rate and $Q^{\prime }$ is a new value function. The actor network updates the parameter θ using chain differentiation (Equation 9).


(9) $$\nabla_{\theta }J\left(\theta \right)=\frac{1}{n}\overset{n}{\underset{i=1}{\sum }}\left[\nabla Q\left(s_{i},a_{i}\right)\nabla \pi_{\theta }\left(a_{i}|s_{i}\right)\right]$$

The critical network updates the parameter w, by taking the mean square error (MSE) between the expected and actual values, i.e, as represented in Equation 10.


(10) $$J\left(w\right)=\frac{1}{n}\overset{n}{\underset{t=1}{\sum }}{\left(Q\left(s^{\prime },a^{\prime },w^{\prime }\right)-Q\left(s_{i},a_{i},w\right)\right)}^{2}$$

here, $Q\left(s^{\prime },a^{\prime },w^{\prime }\right)$ is the target value calculated by the critic target network.

### 3.2 Reward function design

The quality of the reward function is a key factor affecting the results of the model. Intelligent agents for a single task have clear reward goals, so it is necessary to maximize the reward value. However, in dealing with complex autonomous driving tasks, it is difficult to have a single clear reward objective. Therefore, this paper intends to design a reward function through a combination approach, known as:

1) Path tracking capability. The lateral distance between the robot’s center of mass position $y_{i}$ and the expected trajectory $y_{j}$ was designed to describe the tracking accuracy of the robot as in Equation 11:


(11) $$R_{1}=\Delta_{y}=\left|y_{i}-y_{j}\right|$$

The ratio of tracking accuracy error to allowable error is ∆_1_, and its mentioned in Equation 12.


(12) $$\Delta_{1}=\Delta_{y}/0.3$$

2) Speed. $R_{2}=V_{x}\cos\left(\theta \right)$ , where, $V_{x}\cos\left(\theta \right)$ is the speed of the vehicle along the expected path direction, and it is expected to complete the driving task quickly in limited time and safety.

3) Robot stability. The stability of a robot is mainly reflected by its yaw rate and center of mass lateral deviation angle. The yaw rate is $R_{3}=\Delta_{\omega }=\left|\omega_{p}-\omega_{t}\right|$ and described as the difference between the actual yaw rate $\omega_{p}$ and the expected yaw rate $\omega_{t}$ , where, $\omega_{t}=\mathrm{mim}\left(\left|\omega_{des}\right|,\omega_{d}\right)sgn\left(\delta \right)$ and $\omega_{d}$ is the upper limit of lateral angular velocity. $\omega_{des}$ is the yaw rate under steady-state steering, and $\omega_{des}=G_{\mathrm{\omega zss}}\times \delta$ . Here, $G_{\mathrm{\omega zss}}$ is known as steady-state gain of the yaw rate and δ is the angle of the steering wheel.

The ratio of lateral angular velocity error to expected angular velocity is $\Delta_{2}=\Delta_{\omega }/\omega_{t}$ . Similarly, the center of mass deviation angle is described by the difference between the actual center of mass deviation angle $\beta_{p}$ and the expected center of mass deviation angle $\beta_{t}$ as represented in Equations 13, 14.


(13) $$R_{4}=\Delta_{\beta }-\left|\beta_{p}-\beta_{t}\right|$$

(14) $$\beta_{t}=\min\left(\left|\beta_{des}\right|,\beta_{d}\right)sgn\left(\delta \right)$$

where, $\beta_{d}$ is the upper limit of the lateral deviation angle of the center of mass. $\beta_{des}$ is the lateral deviation angle of the center of mass under steady-state turning, and $\beta_{des}=G_{\mathrm{\beta zss}}\times \delta$ . $G_{\mathrm{\beta zss}}$ is the steady-state gain of the center of mass sideslip angle.

The ratio of the deviation angle of the center of mass to the expected deviation angle of the center of mass is $\Delta_{3}=\Delta_{\beta }/\beta_{t}$ .

4) Steering stability. The smoothness of steering represents the degree of steering wheel oscillation, and a coefficient of variation $R_{5}$ is $R_{5}=C_{v}=\sigma /\tilde{\theta }$ . Where, σ is the standard deviation of the steering wheel angle and $\tilde{\theta }$ is the average value of the steering wheel angle. The aforementioned RS-DDPG approach involves a collective reward shaping that is distributed across all agents in collaborative circumstances. Nevertheless, this factor is frequently ignored in several real-world scenarios.

### 3.3 Adaptive weight design

The accuracy of path tracking and the stability performance of robots have a significant impact on the control of autonomous driving path tracking. When both cannot be met simultaneously, it is necessary to determine which indicator with a large gap should be dealt with first. This study designed adaptive weight coefficients. When the percentage of tracking accuracy error is greater than the percentage of stability error, the weight of the reward function for tracking accuracy will increase, and vice versa. The weight and stability weight coefficients for tracking accuracy are in Equations 15, 16


(15) $$C_{1}=0.5+e^{\Delta_{1}}/\left(e^{\Delta_{1}}+e^{\Delta_{2}+\Delta_{3}}\right)$$

(16) $$C_{2}=0.5+e^{e^{\Delta_{2}+\Delta_{3}}}/\left(e^{\Delta_{1}}+e^{\Delta_{2}+\Delta_{3}}\right)$$

The tracking accuracy weight coefficient and stability weight coefficient satisfy $C_{1}=C_{2}=2$ expressions. During the training process, AVs may encounter two situations: normal driving and exceeding the lane. The reward function for normal driving has been designed, and the situation of exceeding the lane is uniformly set to 0 here. The expression for the reward function is represented in Equation 17:


(17) $$R=\{\begin{array}{c} R_{2}-C_{1}R_{1}-C_{2}\left(R_{3}+R_{4}\right)-R_{5},\mathrm{Normal}\\ 0,\;\mathrm{Beyond the lane}\;\; \end{array}$$

## 4 Simulation testing and analysis

In order to evaluate the advantages and disadvantages of the proposed autonomous driving robot control method in this study, a model will be built on the Apollo simulation platform, and the trajectory tracking process of intelligent robots will be simulated and analyzed using proposed algorithm and actual DDPG algorithm. The RS-DDPG algorithm in this study is based on the actor-critic network structure, where the actor network is updated using the policy gradient method, and the policy is optimized in a better direction based on the policy gradient. The input of the actor network is observation (position, angle, speed, etc.), and the output is control signals, such as steering wheel angle and accelerator brake.

**Figure 7:**
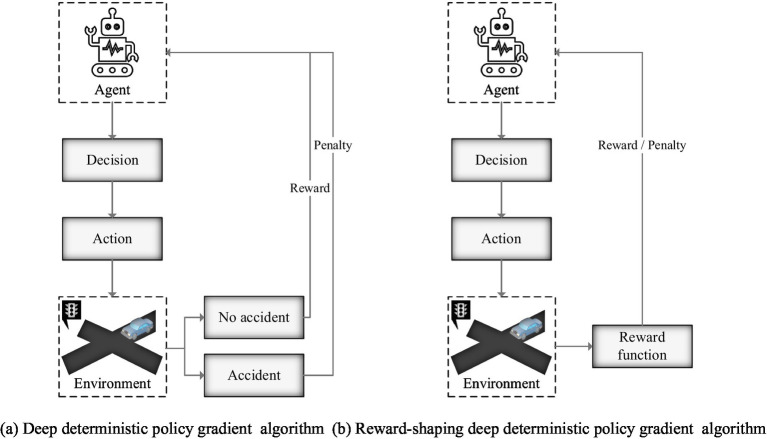
Evaluation methods of DDPG and RS-DDPG algorithms.

The critic network develops using the behavioral value function, which takes state and behavior as input factors and outputs return values as output variables. This network is utilized to assess the efficiency of different techniques. The paper’s RS-DDPG approach is more generalizable and robust than the traditional DDPG method since it uses a novel reward function. The evaluation methods of the DDPG algorithm and RS-DDPG algorithm are shown in Figure 7.

**Figure 8:**
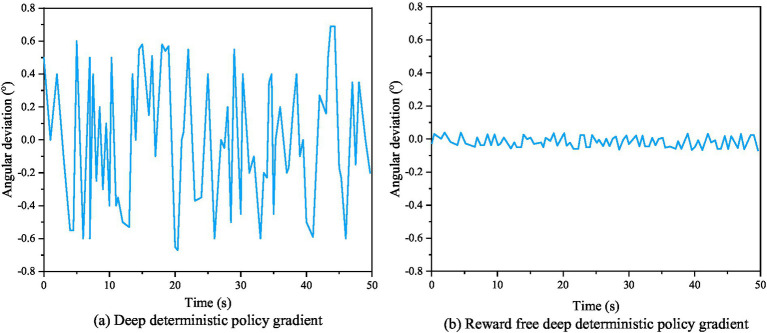
Heading angle deviation curves of autonomous robots before and after algorithm improvement.

**Figure 9:**
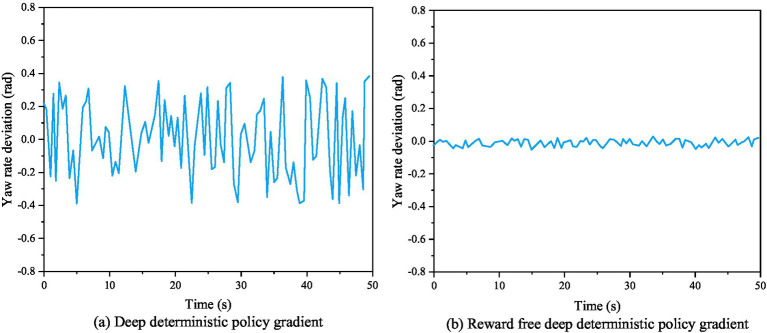
Yaw rate deviation curve of autonomous robots before and after algorithm modification.

**Figure 10:**
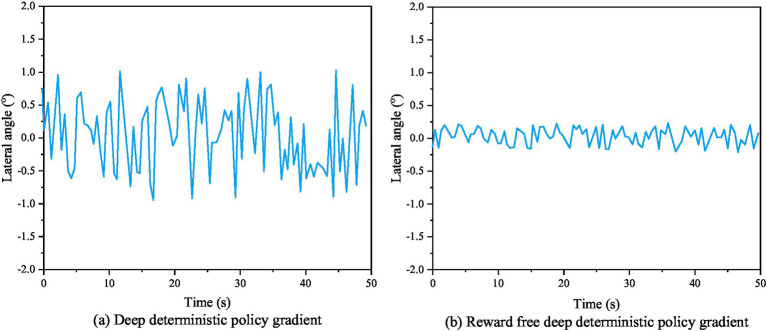
Curve of center of mass lateral deviation angle of autonomous driving robots before and after algorithm enhancement.

Figure 7A shows the actual evaluation algorithm. It is noticeable that the actual algorithm differentiates between evaluation techniques for intelligent robots that are based on accidents and non-accidents. However, the trained results failed to achieve the required accuracy standards for intelligent robots path tracking. Figure 7B shows the enhanced assessment achieved using a combined method in designing a reward function, resulting in a more logical assessment and better accuracy of the control effect after training. The heading angle deviation curve, yaw rate deviation curve, and center of mass lateral deviation curve of AVs before and after algorithm improvement are shown in Figures 8–10, respectively.

**Figure 11:**
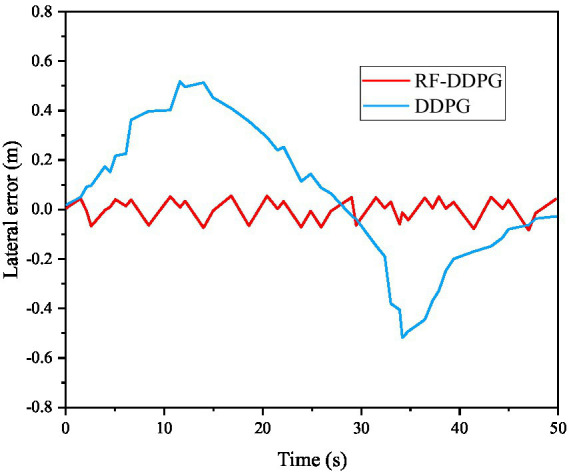
Comparison of lateral errors of autonomous robots before and after algorithm improvement.

From Figures 8–10, it can be seen that the stability performance of AVs using RS-DDPG control algorithm during the experimental process is significantly higher than that of robots using DDPG algorithm, and the control process is more reasonable. This study not only confirms the efficacy of the algorithm strategy output, but also demonstrates the strong application of the improvement approach in the simulated environment. Figure 11 shows a comparison of lateral errors between AVs using RS-DDPG and DDPG algorithms.

As shown in Figure 11, the comparison of lateral errors between AVs using RS-DDPG and DDPG approaches can also be seen intuitively that the RS-DDPG control algorithm has higher tracking accuracy performance than the DDPG algorithm, and the control process is more reasonable. Table 2 compares the results of different tracking control values using DDPG and RS-DDPG control algorithms. From the data in Table 2, it can be concluded that the tracking control values of RS-DDPG algorithm are better than the corresponding values of DDPG algorithm.

**Table 2:** Comparison of tracking control values before and after algorithm improvement.

Parameters	Tracking control values for different algorithms	
	DDPG	RS-DDPG
Maximum absolute value of lateral error (*m*)	0.52	0.07
Average absolute value of lateral error (*m*)	0.22	0.03
Maximum absolute value of angular deviation (°)	0.60	0.05
Average absolute value of angular deviation (°)	0.29	0.03
Maximum absolute value of angular velocity deviation (rad)	0.40	0.03
Average absolute deviation of angular velocity (rad)	0.22	0.01

## 5 Conclusion

This article takes intelligent robots as the research object and uses reinforcement learning based methods to study the optimal control problem of robots in tracking trajectories. A DRL based RS-DDPG and visual SLAM path tracking algorithms are proposed, aiming to optimize the tracking accuracy and operational stability of robots. Enhanced the robustness of the visual SLAM system in dynamic environments, and utilized semantic information to generate a static semantic octree map, saving a lot of storage space. At the same time, the generated map can be directly used for robot path planning. On the basis of DRL, these algorithm designs a reward function and adaptive weight coefficients for intelligent robots in trajectory tracking, thereby optimizing the parameters of RS-DDPG. The controller takes the current position, speed, tracking path information, and heading angle of the robot as inputs, and outputs the steering wheel angle and throttle brake. Intelligent robot trajectory tracking performance using the algorithm proposed in this paper and the actual DDPG algorithm was tested on a simulation platform. The simulation results prove that the RS-DDPG based RL method, proposed in this paper, has substantial enhancements in tracking accuracy and control effectiveness compared to the actual DDPG method. Furthermore, it guarantees the safety and stability of the robot’s driving process. To explore the problem of intelligent robot trajectory tracking further, the next research will continue to conduct trajectory planning, apply the following control strategies to the planned trajectory and conduct simulation verification of the trajectory tracking strategy. On this basis, the RS-DDPG algorithm can be further improved to enhance its control accuracy and robustness. This study is of great significance for intelligent robots’ autonomous driving and intelligent transportation systems’ development. It is expected to provide effective technical support for achieving safe driving of robots and smooth traffic.

## Data Availability

The original contributions presented in the study are included in the article/supplementary material, further inquiries can be directed to the corresponding author.
